# Biases in probabilistic category learning in relation to social anxiety

**DOI:** 10.3389/fpsyg.2015.01218

**Published:** 2015-08-17

**Authors:** Anna Abraham, Christiane Hermann

**Affiliations:** ^1^School of Social, Psychological and Communication Sciences, Leeds Beckett University, LeedsUK; ^2^Department of Clinical Psychology, Justus Liebig University of Giessen, GiessenGermany

**Keywords:** social anxiety, positive versus negative feedback, probabilistic learning, reinforcement learning, instrumental learning, information processing biases

## Abstract

Instrumental learning paradigms are rarely employed to investigate the mechanisms underlying acquired fear responses in social anxiety. Here, we adapted a probabilistic category learning paradigm to assess information processing biases as a function of the degree of social anxiety traits in a sample of healthy individuals without a diagnosis of social phobia. Participants were presented with three pairs of neutral faces with differing probabilistic accuracy contingencies (A/B: 80/20, C/D: 70/30, E/F: 60/40). Upon making their choice, negative and positive feedback was conveyed using angry and happy faces, respectively. The highly socially anxious group showed a strong tendency to be more accurate at learning the probability contingency associated with the most ambiguous stimulus pair (E/F: 60/40). Moreover, when pairing the most positively reinforced stimulus or the most negatively reinforced stimulus with all the other stimuli in a test phase, the highly socially anxious group avoided the most negatively reinforced stimulus significantly more than the control group. The results are discussed with reference to avoidance learning and hypersensitivity to negative socially evaluative information associated with social anxiety.

## Introduction

Social anxiety refers to the feelings of uneasiness that emerge during social interactions from being judged by other people. As the hallmark symptom of social anxiety disorder or social phobia is a disproportionate fear of negative evaluation, an attentional bias for negative social cues, such as angry facial expressions or averted eye gaze, are posited to lie at the root of this disorder given that these are overt indicators of social threat or rejection (Clark and Wells, 1995; Rapee and Heimberg, 1997). Evidence supporting this notion comes from both behavioral and neuroscientific studies (Mogg et al., 2004; Sewell et al., 2008).

The idea that there is enhanced attention or “hypervigilance” to social threat information in clinical and subclinical social anxiety has received tremendous support. For instance, when comparing low and high socially anxious groups (HSA) in involuntary (exogenous) versus voluntary (endogenous) direction of attention when processing threatening stimuli, Moriya and Tanno (2011) found that the exogenous attention system is biased toward angry faces among highly socially anxious individuals (Moriya and Tanno, 2011). But this bias for hypervigilance in social anxiety is a temporally limited phenomenon as it is restricted to the first 100–500 ms following the presentation of threatening stimuli (Staugaard, 2010).

Alongside the evidence for hypervigilance in relation to social anxiety, researchers have also documented “avoidance” strategies in the presence of social cues (Heinrichs and Hofmann, 2001). For instance, high socially anxious individuals display diminished attentional allocation or attentional biases away from positive social stimuli, such as words reflecting positive social evaluations (Taylor et al., 2010).

The seemingly diverging findings of both hypervigilance toward and avoidance of negative social cues have been accommodated into a common framework (Mogg et al., 1997; Amir et al., 1998) where social anxiety is held to be characterized by hypervigilance during the initial phase of information processing of threatening social stimuli followed by avoidance of such stimuli at a later phase. The impact of such attentional biases have been investigated in the context of learning mechanisms in social anxiety, particularly in terms of conditioned fear (Hermann et al., 2002; Gros et al., 2009; Ly and Roelofs, 2009; McTeague et al., 2009; Lissek, 2012; Pejic et al., 2013). Lissek et al. (2008), for instance, reported that a group of participants with social anxiety disorder compared to an unaffected control group demonstrated elevated fear conditioning in response to negative facial expressions. This was evidenced by a potentiation in the startle-blink reflex when angry faces were the conditioned stimuli compared to neutral or happy facial expressions. As the group differences were not dependent on levels of anxiety elicited by the unconditioned stimulus, associative processes appeared to be the active mechanism underlying the enhanced conditioned startle-potentiation among the individuals with social anxiety disorder (Lissek et al., 2008).

Although clinically relevant information processing biases in associative processing can also be assessed using instrumental learning paradigms, few studies have explored this avenue in the case of social anxiety (Cavanagh et al., 2011b; Button et al., 2012; Stevens et al., 2014). The learning process in Pavlovian learning or classical conditioning refers to the elicitation of an innate response to a neutral stimulus that comes to occur as a consequence of repeated pairings of the neutral stimulus with a potent stimulus, which automatically elicits that innate response. Operant conditioning or instrumental learning differs in that the learning process reflects how a response is shaped by a reinforcing stimulus that follows it, with positive consequences leading to strengthening of the response and negative outcomes leading to a weakening of the response. One important rationale behind evaluating instrumental learning in the context of clinical disorders is that the focus in classical conditioning is either on positive or on negative learning effects, but not the integrated effect of both together. Being able to incorporate both positive and negative elements of the complex learning processes and to study how individuals make appropriate responses within a single paradigm enables a more nuanced approach to understanding information processing biases.

Probabilistic instrumental learning paradigms allow for the investigation of how stimulus–response associations are learnt through trial-and-error, and provide the opportunity to evaluate the factors that serve as modulators in such situations. We take decisions on a regular basis about how to optimally respond in any given situation, and the strategies we adopt in taking these decisions are guided by our previous experiences of the relations and contingencies between events, actions, and outcomes. Probabilistic paradigms are more ecologically valid than their deterministic counterparts as uncertainty about the contingencies between events, actions, and outcomes are intrinsic to our environment.

In probabilistic categorization learning, the same stimuli belong to multiple categories of varying probabilities. As a result, a response to stimulus can be reinforced as either correct or incorrect on a probabilistic basis and this feedback drives category learning (Craig and Baron-Cohen, 1999; Kruschke and Johansen, 1999; Ashby and Maddox, 2005). While learning is abetted by both positive action outcomes (leading to repetition of successful behavior) and negative action outcomes (leading to avoidance of erroneous behavior), some studies have shown that the degree to which one learns from successes versus errors varies across individuals as a function of genetic makeup (Klein et al., 2007; Frank and Hutchison, 2009), neurological or psychiatric disorders (Chase et al., 2010; Averbeck et al., 2011; Cavanagh et al., 2011a; Endrass et al., 2011; Shiner et al., 2012), pharmacological manipulations (Frank et al., 2004), or even interventions using brain stimulation (Ott et al., 2011). Reinforcement learning models have been applied to understand the nature of psychological dysfunctions associated with several psychiatric disorders, including attention-deficit/hyperactivity disorder (ADHD) and schizophrenia (Maia and Frank, 2011).

The objective of the current study was to assess potential information processing biases associated with heightened levels of social anxiety in a non-clinical sample. Volunteers were selected to participate in the study based on their scores on a self-report scale, that they filled out during a pre-screening session, which assessed their fear of being evaluated negatively, the central cognitive symptom of social anxiety^1^. We expected group-based differences in instrumental learning based on the aforementioned literature on conditioned fear, but the nature or direction of this difference was exploratory due to the paucity of research on instrumental learning in social anxiety. A probabilistic category learning paradigm was adapted to this end using socially relevant information as stimuli (neutral faces) and socially evaluative reinforcement as feedback (positive: happy faces, negative: angry faces). Group-based behavioral differences were assessed by comparing performances of a high socially anxious group (HSA) relative to non-socially anxious counterparts (NSA) during learning of stimulus pairs associated with varying probabilistic contingencies (A/B: 80/20, C/D: 70/30, E/F: 60/40). In the learning phase, group based differences were evaluated in terms of the percentages by which high probabilistic stimuli (A, C, E) were chosen relative to low probabilistic stimuli (B, D, F). In the test phase, group based differences were appraised in terms of the percentages associated with choosing the most positively reinforced stimulus (A) and choosing the most negatively reinforced stimulus (B) over all other stimuli.

The rationale in investigating information processing biases in a non-clinical sample associated with a high degree of social anxiety traits follows from the dimensional nature of social anxiety (e.g., Kollman et al., 2006; Crome et al., 2010). Indeed, the evidence suggests that a core cognitive component of social anxiety, namely the fear of negative evaluation, is dimensional in nature (Weeks et al., 2009). Following this line of thought, the study of analogue samples of individuals with a high degree of social anxiety traits has a long tradition in research on cognitive mechanisms and learning processes that are relevant to social anxiety, such as attentional biases (Pineles and Mineka, 2005; Klumpp and Amir, 2009), post-event processing (Dannahy and Stopa, 2007), and avoidance learning (Ly and Roelofs, 2009). Analogue studies are not a substitute for clinical investigations but constitute an alternative subclinical approach that allows investigators to evaluate many assumptions concerning the underpinnings of clinical disorders. The usefulness of combining insights from both clinical and subclinical approaches in order to understand information processing mechanisms that underlie psychiatric disorders has been widely acknowledged (e.g., OCD: Abramowitz et al., 2014; PTSD: Ehring et al., 2011; Anxiety: Tull et al., 2009; Depression: Vredenburg et al., 1993).

## Materials and Methods

### Participants

The sample selection was carried out by screening 280 undergraduate student volunteers enrolled at the University of Giessen in Germany who completed the German version of the Fear of Negative Evaluation Scale (FNE; Watson and Friend, 1969; Vormbrock and Neuser, 1983; *M* = 45.26, SD = 10.01). The FNE is a widely employed social anxiety measure, which assesses the degree of fear toward criticism (e.g., “I often worry that I will say or do the wrong things”). On the basis of their FNE scores, 80 participants were selected and assigned to one of two groups for the main study. The HSA included participants who obtained an FNE score greater than the Mean + 1 SD while the NSA included those who obtained a score between Mean ± 1 SD^2^.

All participants were native German speakers with no reported history of neurological or psychiatric illness (telephone screening prior to recruitment). None were taking medication at the time of measurement. All gave informed consent before participation and received either payment (EUR 8) or course credits for their participation. The experimental standards of the study were approved by the Local Ethics Committee of the Justus Liebig University of Giessen in Germany.

After excluding participants who did not meet the inclusion criteria for data analysis (details provided below in the subsection on Exclusion Criteria), the final sample included 62 participants within two subgroups: HSA (*n* = 30; 17 males; mean age = 22.5; age range = 19–29) and NSA (*n* = 32; 15 males; mean age = 22.59; age range = 19–27). The HSA and NSA groups were not significantly differentiable in terms of age (*t*_60_ = 0.16, *p* = 0.87) or gender (χ^2^ = 0.59, *p* = 0.46). All the reported statistical analyses below were carried out on this final sample.

Following the experiment, all participants completed the German versions of the Social Phobia Scale (SPS) and Social Interaction Anxiety Scale (SIAS; Mattick and Clarke, 1998; Stangier et al., 1999). The SPS and SIAS assess the fear of being observed during routine tasks and general social interactions (e.g., “I have difficulty talking with other people”). The descriptive data for the self-report measures across all groups are given in **Table 1**. The HSA were found to have significantly higher scores than the NSA on all the three measures of social anxiety: the FNE (*t*_60_ = 12.23, *p* < 0.001, Cohen’s *d* = 3.13), the SPS (*t*_60_ = 5.67, *p* < 0.001, *d* = 1.43), and the SIAS (*t*_60_ = 6.34, *p* < 0.001, *d* = 1.6), indicating that the HSA group was significantly more socially anxious than the NSA group. There were no gender differences across any of the social anxiety measures (all *p* > 0.1), and assessing the reliability coefficient of the scales within the sample revealed a high degree of internal consistency (FNE^3^: Cronbach’s α = 0.717; SPS: Cronbach’s α = 0.898; SIAS: Cronbach’s α = 0.892).

**Table 1:** Descriptive data of all the self-report dependent measures.

	HSA		NSA		All	
	Mean	SD	Mean	SD	Mean	SD
N (Female: Male)	30 (13:17)		32 (17:15)		62 (30:32)	
Age	22.50	2.43	22.59	2.15	22.55	2.27
FNE	62.00	4.81	42.94	7.16	52.16	11.37
SPS	23.90	11.07	10.75	6.82	17.11	11.21
SIAS	30.93	11.79	15.28	7.27	22.85	12.45
Angry (SAM Valence)	2.53	1.38	3.16	1.72	2.85	1.59
Neutral (SAM Valence)	4.54	0.81	4.51	0.86	4.52	0.83
Happy (SAM Valence)	7.27	1.72	7.13	1.84	7.19	1.77
Angry (SAM Arousal)	6.77	1.77	6.16	1.87	6.45	1.84
Neutral (SAM Arousal)	3.80	0.73	3.50	1.00	3.64	0.89
Happy (SAM Arousal)	4.50	2.36	3.75	1.87	4.11	2.14

*FNE, Fear of Negative Evaluation Scale; HSA, high social anxiety group; NSA, non-socially anxious group; SAM, Self-Assessment Manikin Ratings of the Face Stimuli; SIAS, Social Interaction Anxiety Scale; SPS, Social Phobia Scale.*

### Experimental Task

The probabilistic category selection task was adapted for use in the current study (Frank et al., 2004). The difference that was instantiated in the present paradigm was that faces were employed as the learning stimuli (neutral faces) and feedback stimuli (happy and angry faces; Lundqvist et al., 1998). The selection of faces was based on ratings of an unpublished pilot study where the faces with best match to the emotions being portrayed were selected. In line with the protocol used a previous study on learning (Stevens et al., 2014), male faces were employed as learning and feedback stimuli for the female participants and vice versa. The experiment was separated into two phases: a learning phase and a test phase. Both phases were programmed using Presentation^®^ software (www.neurobs.com), which was also used to present the stimuli (size: 270 × 220) (resolution: 800 × 600) via a computer screen (resolution: 800 × 600). Participants were seated 60 cm away from the screen to perform the task (visual angle: 14.26° × 5.53°).

**FIGURE 1:**
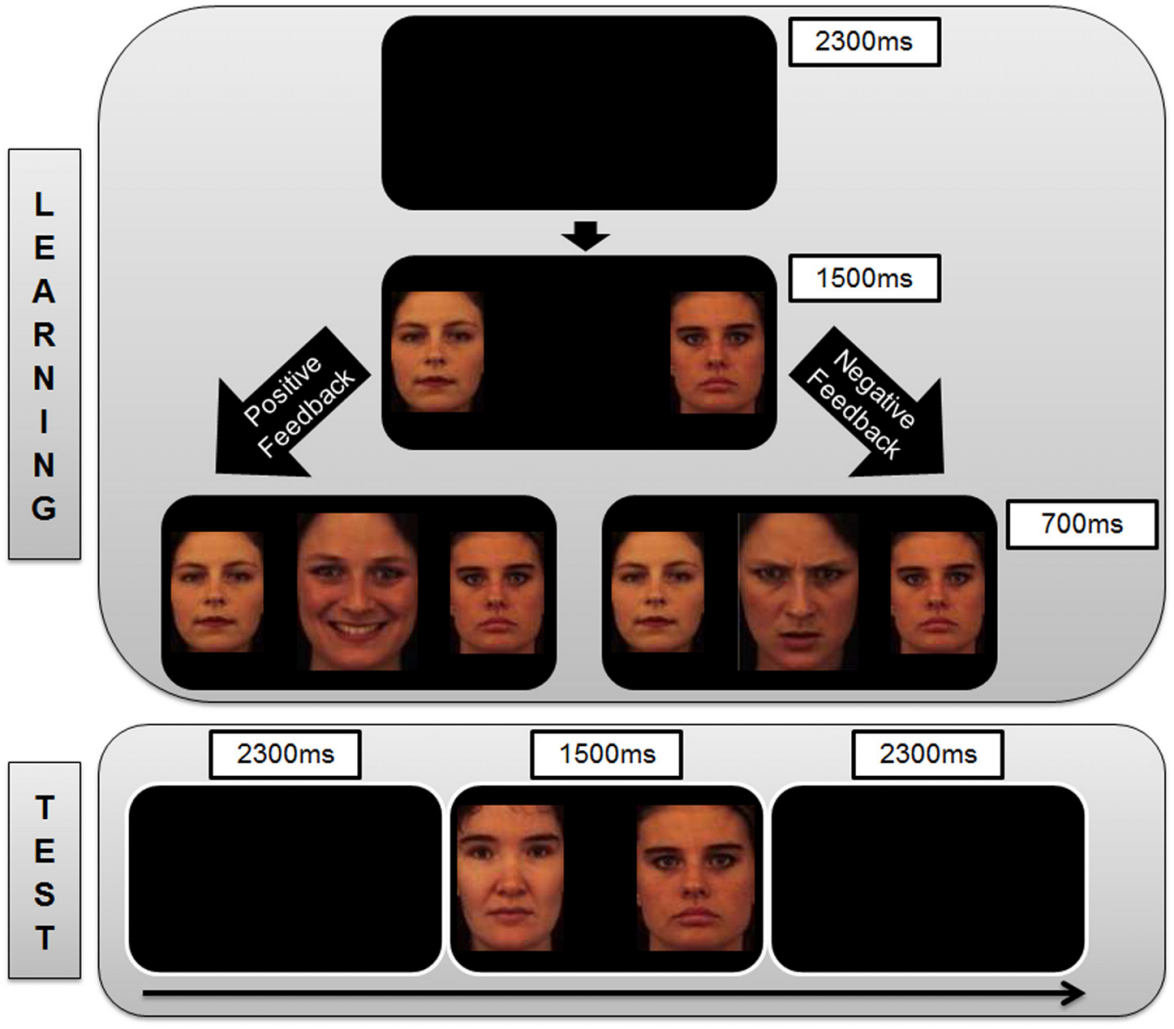
**Sequence of trial events. (Top)** The representation of trial events in the Learning Phase of the study. Each trial begins with a blank screen which is presented for 2300 ms, followed by a pair of neutral face stimuli for 1500 ms. Subjects are instructed to indicate with a key press (left or right) which of the two faces is the correct option in the current trial. Based on the accuracy of the subject’s choice, s/he is given positive feedback (happy face) or negative feedback (angry face) that is presented for 700 ms between the neutral face stimuli. If the subjects take too long to respond, they receive feedback in the form of a message between the neutral face stimuli stating “Oops! Too Late” (not shown in the Figure). This is followed by the presentation of a blank screen (2300 ms) which marks the commencement of the next trial. **(Bottom)** The representation of trial events during the Test Phase of the study. Each trial begins with a blank screen which is presented for 2300 ms, followed by a pair of neutral face stimuli for 1500 ms. Subjects are instructed to indicate with a key press (left or right) which of the two presented neutral faces is the correct option. No feedback on the responses is given within the test phase. This is followed by the presentation of a blank screen (2300 ms) which marks the commencement of the next trial.

In the learning phase, subjects were presented with one of three different stimulus pairs of neutral faces (Face A and Face B, Face C and Face D, Face E and Face F) in each trial (140 trials per pair). The subjects were instructed to learn which one of the faces within each stimulus pair (AB, CD, EF) was associated with more positive feedback and to optimize their performance to be as accurate as possible in making their responses. The three stimulus pairs were presented in pseudo-randomized order (e.g., equivalent occurrence of all trial transition types). The subjects had to select one of the two stimuli that were presented on each trial by pressing the appropriate response key on the keyboard using the index and middle fingers of the right hand (left arrow key to select the face presented on the left of the screen, right arrow key to select the face presented on the right of the screen). After they had indicated their choice, feedback immediately followed to indicate whether this choice was correct (in the form of a happy face) or incorrect (in the form of an angry face) for that trial (**Figure 1**). The identities of the faces used as the experimental stimuli (neutral faces A – F) were distinct from those of the feedback stimuli (happy and angry faces).

This feedback was probabilistic in that in AB trials, the choice of stimulus A led to positive feedback in 80% of the trials and negative feedback in 20% of the trials. Choosing B therefore led to negative feedback in 80% of the trials and positive feedback on 20% of the trials. CD and EF pairs were less reliable as choosing C over D led to positive feedback in 70% of the trials and negative feedback in 30% of the trials. Choosing E over F led to positive feedback in 60% of the trials and negative feedback in 40% of the trials. The probabilistic contingencies associated with each of the three pairs were unknown to participants in advance. They acquired this knowledge over the learning phase in which they learned, through feedback on their responses, to choose the A, C, and E high positive feedback probability stimuli (or the low negative feedback probability stimuli) more often than the B, D, and F low positive feedback probability stimuli (or the high negative feedback probability stimuli).

Participants learned about these probability contingencies through positive feedback (e.g., seeing a happy face more often when choosing A) and negative feedback (e.g., seeing an angry face more often when choosing B). A test phase was carried out to determine if participants learned more from the positive or negative consequences of their responses by presenting them novel combinations of stimulus pairs that were not encountered during the learning phase. The A and B stimuli were presented in combination with all other stimuli (AC, AD, AE, AF, BC, BD, BE, BF). There were 48 A-trials and 48 B-trials, and no feedback was provided after the choice response during the test phase (**Figure 1**). The percentage of choices for the A stimulus over the others reflected the degree to which participants learned from positive reinforcers as the contingency of positive feedback was highest for the A stimulus. The percentage of avoidance responses toward the B stimulus (i.e., not choosing B) over the others reflected the degree to which participants learned from negative reinforcers as the contingency of negative feedback was highest for the B stimulus.

Following the test phase, participants were required to rate all the faces they were presented with during the experiment (choice stimuli and feedback cues) using the nine-point Self-Assessment Manikin Scale (SAM; Bradley and Lang, 1994) in terms of valence (ranging from 1–9: “extremely pleasant” to “extremely unpleasant”) and arousal (ranging from 1–9: “calm” to “excited”).

### Exclusion Criteria

To ensure that all participants within the final sample were comparable in terms of their behavioral performance, three *a priori* exclusion criteria were applied^4^. The first criterion involved excluding participants who were below average learners. For this purpose, the percentage by which stimulus A (Group Mean_A_ = 76.19, SD_A_ = 14.10) and stimulus C (Group Mean_C_ = 72.99, SD_C_ = 16.96) were selected by each participant in the learning phase were used to discriminate between successful and unsuccessful learners. Any participant whose score lay more than 1.5 SD below the group mean (Cut-off_A_ < 55.04, Cut-off_C_ < 47.55) was classified an unsuccessful learner and was excluded from the final sample. Thirteen participants (HSA = 6, NSA = 7) were excluded from the original pool of subjects (*n* = 80) on the basis of this first criterion^5^.

The second criterion was based on the SAM valence ratings of the stimuli where subjects were excluded if they rated the happy faces to be more negative than the angry or neutral faces and/or the angry faces to be more positive than the happy or neutral faces. This is because, in such situations, it could not be claimed that the participants experienced positive feedback when presented with the happy face or negative feedback when presented with the angry face. Five participants (HSA = 2, NSA = 3) were excluded from the original pool of subjects on the basis of this criterion.

The third criterion was that any participant who had more than 10% misses (no response made within the allotted 1500 ms) during the learning phase would be excluded as a high proportion of misses indicates inattentiveness during the experiment. None of the participants were excluded on the basis of this criterion.

### Statistical Analyses

Repeated measures ANOVAs were used to determine (a) the optimization of the stimulus characterization, (b) which variables influenced choice patterns in the learning phase, and (c) which variables influenced choice patterns in the test phase. *T*-tests were used for planned pairwise and independent samples contrasts. Effect sizes are also reported – partial eta-squared for the ANOVAs and Cohen’s *d* for the *t*-tests.

## Results

The descriptive data associated with the groups across all variables are displayed in **Table 1** (stimulus characterization), **Table 2** (learning phase) and **Table 3** (test phase)^6^.

**Table 2:** Descriptive data of dependent measures from the learning phase of the experiment.

	HSA		NSA	
	Mean	SD	Mean	SD
Percentage chosen (over all trials)*				
A (80% correct)	77.10	11.94	81.76	11.17
B (20% correct)	18.36	9.50	15.11	10.12
C (70% correct)	76.07	14.33	76.29	11.01
D (30% correct)	19.81	11.62	21.05	10.42
E (60% correct)	69.40	15.72	76.68	14.40
F (40% correct)	26.90	13.56	21.00	13.79
**Percentage chosen difference**				
B–A (60% difference)	58.74	21.01	66.65	21.18
D–C (40% difference)	56.26	25.51	55.25	21.33
F–E (20% difference)	42.49	28.97	55.67	28.15

*Please note that the percentage chosen within each stimulus pair (AB, CD, EF) may not sum to 100% due to the fact that there were some misses (responses that were too slow and outside the response window). HSA, high socially anxious group; NSA, non-socially anxious group.

**Table 3:** Descriptive data of dependent measures from the test phase of the experiment.

	HSA		NSA		
	Mean	SD	Mean	SD	Significance (two-tailed)
Choose A	51.25	27.29	62.63	28.15	0.112
Avoid B	74.72	20.37	64.19	18.88	0.039

*HSA, high socially anxious group; NSA, non-socially anxious group.*

### Stimulus Characterization

**FIGURE 2:**
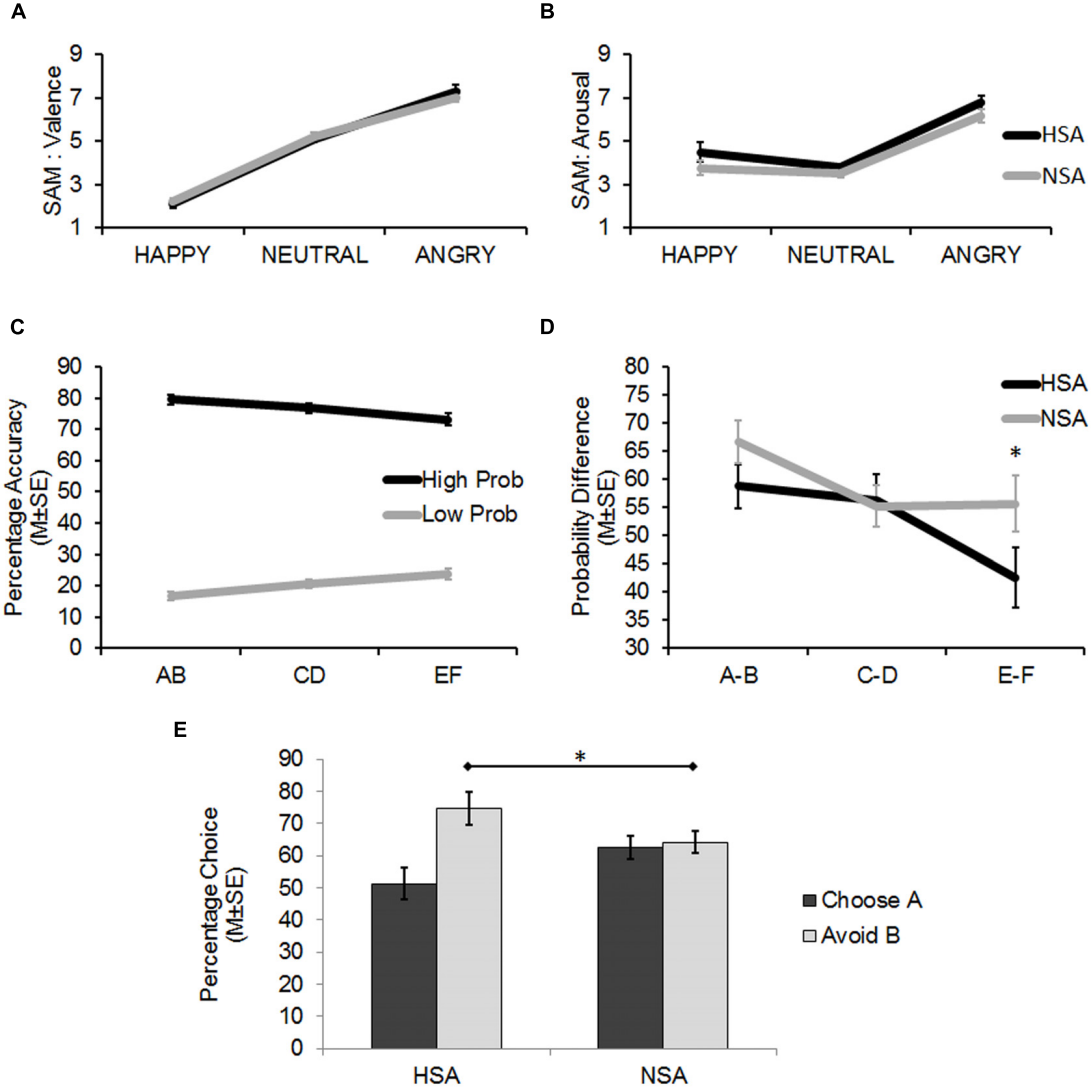
**(A)** Findings from the self-assessment manikin (SAM) ratings (Mean ± 1 SE) of the face stimuli on Valence (pleasant–unpleasant). Angry faces were rated to be significantly more unpleasant than the happy and neutral faces, and neutral faces were rated as significantly more unpleasant than happy faces (all *p* < 0.001). **(B)** Findings from the SAM ratings (Mean ± 1 SE) of the face stimuli on Arousal (calm to excited). Angry faces were rated to be significantly more arousing than the happy and neutral faces (all *p* < 0.001). The HSA reported a generally higher level of arousal when viewing the face stimuli than the NSA (*p* < 0.05). **(C)** Results from the Learning Phase showing successful learning (percentage accuracy: Mean ± 1 SE) of the probabilistic task. On being presented with the three probabilistic pairs (AB, CD, EF), the high probability stimuli (A, C, E) within each pair (dark line) were selected more often than their corresponding low probability stimuli (B, D, F; light line). Differences in probability contingencies of the stimuli were also successfully learnt (A > C > E > F > D > B). **(D)** Results from the Learning Phase (Mean ± 1 SE) showing that the HSA more accurately detected the actual probability difference between the EF pair than the NSA. **(E)** Results from the Test Phase (Mean ± 1 SE) showing that the HSA group avoided B, the most punished stimulus, significantly more than the NSA (*p < 0.05).

The 3 × 2 ANOVA (*stimulus:* positive/neutral/negative × *group*: HSA/NSA) for the SAM ratings of stimulus-associated valence revealed a significant main effect (linear trend) for stimulus (*F*_1,60_ = 205.21, *p* < 0.001; $\eta_{p}^{2}$ = 0.77). Angry faces were rated as significantly more negative than neutral and happy faces, while neutral faces were rated as significantly more negative than happy faces (planned pairwise contrasts: all *t*_61_ > 7.73, *p* < 0.001, *d* > 1.32; **Figure 2A**).

The 3 × 2 ANOVA (*stimulus*: positive/neutral/negative × *group*: HSA/NSA) for the SAM ratings of stimulus-associated arousal revealed a significant main effect (quadratic trend) for stimulus (*F*_1,60_ = 59.88, *p* < 0.001; $\eta_{p}^{2}$ = 0.50; **Figure 2A**). Angry faces were rated as significantly more arousing than neutral and happy faces (planned pairwise contrasts: all *t*_61_ > 7.22, *p* < 0.001, *d* > 1.18). However, happy faces were not rated as being significantly more arousing than neutral faces (*t*_61_ = 1.61, *p* = 0.113, *d* = 0.28).

While no interaction effects were found across both analyses, one revealed a significant main effect for group (*F*_1,60_ = 4.14, *p* = 0.046; $\eta_{p}^{2}$ = 0.065) on the dependent variable of stimulus-associated arousal, which indicated that the HSA reported significantly more arousal than the NSA when viewing the face stimuli in general, regardless of the associated valence (**Figure 2B**).

In sum, the analyses revealed an optimal stimulus characterization such that angry faces were appraised as being more negative than happy and neutral faces, and neutral faces were appraised as being more negative than happy faces (angry > neutral > happy). Angry faces were also appraised as being more arousing than happy and neutral faces (angry > neutral, angry > happy). In addition, the HSA experienced generally higher levels of arousal when viewing the face stimuli compared to the NSA.

### Experiment: Learning Phase

The 3 × 2 × 2 ANOVA (*probabilistic pair*: AB/CD/EF × *probability*: high/low × *group*: HSA/NSA) on percent chosen revealed significant main effects for probabilistic pair (*F*_1,60_ = 9.4, *p* = 0.003; $\eta_{p}^{2}$ = 0.135) and probability (*F*_1,60_ = 503.52, *p* < 0.001; $\eta_{p}^{2}$ = 0.894) alongside a significant two-way linear interaction effect for probabilistic pair × probability (*F*_1,60_ = 17.21, *p* < 0.001; $\eta_{p}^{2}$ = 0.223). Together these findings indicated that learning had successfully taken place as high positive feedback probability stimuli were selected more often than low positive feedback probability stimuli. Moreover, the differences in probability contingencies of each of the stimuli across the probabilistic pairs were also successfully learnt (**Figure 2C**: A:80 > C:70 > E:60 > F:40 > D:30 > B:20, planned pairwise contrasts: all *t*_61_ > 1.08, *p* < 0.076).

There was also a significant three-way quadratic interaction effect for probabilistic pair × probability × group (*F*_1,60_ = 4.25, *p* = 0.044; $\eta_{p}^{2}$ = 0.066) on percent chosen. This interaction was driven by trends for the HSA group (E: 69%, F: 27%) to more accurately estimate the probability contingency corresponding to the E (*t*_60_ = 1.9, *p* = 0.062, *d* = 0.48) and F (*t*_60_ = 1.7, *p* = 0.095, *d* = 0.43) probabilistic pair (actual contingency: E: 60%, F: 40%) compared to the NSA group (E: 77%, F: 21%).

As the latter was an unexpected finding, further analyses were carried out to assess the sensitivity of the groups in learning the feedback-associated probability contingency differences between the pairs. The actual positive feedback probability difference between probabilistic AB pair is 60% (as 80%–20% = 60%) and those of the CD and EF pairs are 40 and 20%, respectively. By subtracting the percentages by which low positive feedback probability option in each pair was chosen from the high positive feedback probability option (B–A, D–C, F–E), the chosen probability differences between the three pairs was calculated. The between group contrasts revealed that the HSA group tended to more accurately detect the difference between the EF probabilistic pair (*t*_60_ = 1.82, *p* = 0.074, *d* = 0.46) than the NSA group. The HSA and NSA groups were not significantly different in detecting the AB (*p* = 0.145) and CD (*p* = 0.865) probabilistic pair differences (**Figure 2D**).

In summary, the results from the learning phase demonstrated that the probabilistic task with its different probability contingencies across stimulus pairs was successfully learnt by all participants. The HSA tended to be more accurate than the NSA at detecting the actual probability contingency difference between the most ambiguous probabilistic pair: E (60% probability of positive feedback) and F (40% probability of positive feedback).

### Experiment: Test Phase

A 2 × 2 ANOVA (*choice type*: choose A/avoid B × *group*: HSA/NSA) on percent chosen revealed a significant main effect for choice type (*F*_1,60_ = 9.83, *p* = 0.003; $\eta_{p}^{2}$ = 0.141) alongside a significant interaction effect for choice type × group (*F*_1,60_ = 7.53, *p* = 0.008; $\eta_{p}^{2}$ = 0.112). Between group analyses revealed that the HSA and NSA groups did not significantly differ from one another in the degree to which they chose A (51.25 and 62.63%, respectively), the most positively reinforced stimulus in the learning phase, over the other stimuli (*p* = 0.112). However, the HSA avoided B (74.72%), the most negative reinforced stimulus in the learning phase, significantly more often than the NSA (64.19%; *t*_60_ = 2.11, *p* = 0.039, *d* = 0.54; **Figure 2E**; **Table 3**).

## Discussion

The objective of this study was to investigate potential information processing biases in instrumental learning associated with social anxiety. A probabilistic category learning task with socially evaluative feedback was used for this purpose. To ensure the validity of the associated findings, it was necessary to establish that the sample and stimulus characterizations were optimally executed and that the probability contingencies in the experiment were adequately learnt within the training phase. Successful implementation of the experimental paradigm was established on all these counts.

In terms of sample characterization, the high social anxiety group relative to the control group reported experiencing significantly higher levels of social anxiety as well as greater subjective physiological arousal upon viewing faces. This is consistent with other findings in the literature (Westberg et al., 2007). The stimulus characterization analyses revealed that all participants judged the angry faces to be the most unpleasant stimuli, followed by the neutral faces, both relative to the happy faces. Moreover, the whole group analyses from the learning phase of the probabilistic category learning experiment revealed that learning had successfully taken place as the participants’ choices reflected acquired knowledge of the differences between the probability contingencies of all six stimuli (A:80 > C:70 > E:60 > F:40 > D:30 > B:20).

### High Social Anxiety: Evidence for Information Processing Advantages

While the groups did not differ in terms of overall learning performance, the HSA tended to be more sensitive at detecting the actual probabilistic contingencies for the most ambiguous stimulus pair (E/F) during the learning phase than the control group. Specifically, high socially anxious individuals were better at learning the reinforcement contingencies between stimuli that were associated with a high degree of ambiguity given that they manifest relatively subtle differences in terms of the degree of positive-to-negative reinforcers (E: 60% positive, 40% negative; F: 40% positive, 60% negative).

This is a interesting finding that warrants further exploration as it indicates greater sensitivity on the part of the HSA to recognize the actual contingency of positive-to-negative social feedback in highly uncertain or ambiguous contexts. This observation matches well with previous findings that have demonstrated greater accuracy in individuals with social phobia in detecting the actual contingencies between social cues and their associated positive, neutral, or aversive outcomes (Hermann et al., 2004).

A related possibility to consider would be that if the HSA are biased to learn more from socially negative reinforcement, their superior performance on learning the actual contingencies of the E/F pair would be a likely outcome as far more negative feedback is received when responding to this stimulus pair compared to the other less ambiguous pairs. Higher accuracy in estimating the probability of aversive stimuli has, in fact, been reported by Ly and Roelofs (2009). Social phobia patients were better at such estimations during an avoidance task when the avoidance response was no longer available compared to low socially anxious individuals who underestimated the actual probability of the aversive stimulus.

In general though, evidence suggesting the presence of general information processing advantages as a function of social anxiety is somewhat mixed. Some studies have reported poor performance associated with social anxiety, such as in the domain of executive function (Fujii et al., 2013), others report better performance, such as in the domain of working memory (Moriya and Sugiura, 2012), and still others present a mixed picture (Amir and Bomyea, 2011). Amir and Bomyea (2011), for instance, reported that, relative to a control group, patients with generalized social phobia demonstrated comparable verbal working memory capacity for social stimuli but poorer verbal working memory capacity for neutral stimuli. They attributed this dissociation in the findings to the possibility that “enhanced working memory capacity for threat relevant information may be the result of practice with this information.”

On the other hand, a recent study showcased enhanced performance in relation to social anxiety from the domain of visual working memory, where high levels of social anxiety were found to be associated with high visual working memory capacity (Moriya and Sugiura, 2012). However, it should be noted that these advantages in information processing in relation to social anxiety were assessed in terms of general cognitive capacities using socially irrelevant stimuli. In contrast, only socially relevant information (learning and feedback stimuli) were employed in the present study. One avenue for exploration therefore would be to assess whether the apparently heightened sensitivity in learning on the part of the HSA is domain-general (greater accuracy in learning in ambiguous contexts regardless of the type of stimuli or feedback) or domain-specific (greater accuracy in learning of socially relevant information in ambiguous contexts; for a discussion on domain specificity in relation to social anxiety, see Abraham et al., 2013).

### High Social Anxiety: Learning via Greater Sensitivity to Negative Social Cues

The test phase of the probabilistic category learning paradigm was used to assess whether differences would surface in terms of how the groups learned from positive feedback (choosing stimulus A) or negative feedback (avoiding stimulus B). Although differing degrees of social anxiety did not influence overall performance in the learning phase, it exerted a significant impact on the pattern of performance in the test phase. Both groups were not significantly differentiable in terms of the degree to which they chose the most positively reinforced stimulus (A) from the learning phase. However, the HSA avoided the most negative reinforced stimulus (B) from the learning phase significantly more often than the control group. This stimulus was the one with the least favorable odds of positive reinforcement, and hence most strongly associated with the presentation of the angry face. The response pattern of the HSA hence indicated a relatively enhanced propensity to avoid cues that are associated with negative social evaluation.

The complete opposite of this pattern, namely a decreased likelihood to avoid the most negatively reinforced stimulus, using the same probabilistic learning paradigm, albeit with non-social stimuli, has been associated with the presence of the A1 allele of the human genetic polymorphism (DRD2-TAQ-IA; Klein et al., 2007). This allele is associated with a reduction in the D2 dopamine receptor density in the brain, which in turn is related to the manifestation of addictive and compulsive behaviors (but see Anokhin, 2014). So while decreased avoidance of the most negative reinforced stimulus using this kind of probabilistic paradigm has been linked with a reduced sensitivity to negative behavioral consequences (Klein et al., 2007), the present study shows that increased avoidance of the most negative reinforced stimulus is associated with heightened sensitivity to negative social consequences.

Other psychiatric and neurological populations have also been associated with different patterns of activity using probabilistic category learning paradigms. For instance, depression was associated with deficits in reward-based decision making (choosing A) during probabilistic learning as well as a high degree of variability in action selection (Kunisato et al., 2012, but see Chase et al., 2010; Cavanagh et al., 2011a for opposing findings). Unmedicated patients with Parkinson’s disease, in contrast, were found to be better at learning to avoid choices that lead to negative outcomes than learning from positive outcomes. The ingestion of dopamine medication, however, led to a reversal of this response pattern (Frank et al., 2004). Reinforcement learning models are proving to be invaluable in understanding the brain and cognitive dysfunctions associated with several other psychiatric and neurological disorders as well, such as Tourette’s syndrome, ADHD, drug addiction and schizophrenia (Maia and Frank, 2011). The present findings indicate the promise of this approach in unraveling information processing biases that are specific to social anxiety.

The finding of greater avoidance learning in relation to social anxiety fits well with several studies that have shown that social anxiety is related to an increased sensitivity to negative social information. For instance, in comparison to a low socially anxious group, highly socially anxious individuals not only recalled a higher ratio of negative-to-positive images and memories on the Waterloo Images and Memories Interview (WIMI), but also reported poorer episodic detail in the retrieved positive images (Moscovitch et al., 2011). A preference for negative social feedback has also been associated with social anxiety (Valentiner et al., 2011). Indeed, a comparison of high and low speech anxious participants revealed that highly speech anxious individuals displayed an internal attentional bias that was restricted to socially evaluative threat (Mansell et al., 2003).

That this hypersensitivity to negative or ambiguous social information may translate to greater avoidance of the same has also been documented in previous studies. Using a probabilistic Go/No-Go paradigm, Ly et al. (2014), for instance, compared individuals with differing tendencies for social avoidance and found that the high social avoidance group exhibited reduced behavioral inhibition for angry faces relative to happy faces. This failure to inhibit behavior related to aversive social stimuli was held to reflect why socially avoidant individuals perceive situations as more threatening than they are and consequently expect more negative attributions and outcomes. Previous studies have reported enhanced avoidance learning in relation to social anxiety in response to socially ambiguous stimuli, such as neutral faces (Stevens et al., 2014), as well an greater aversive expectancy bias when social situations are ambiguous during avoidance conditioning (Ly and Roelofs, 2009). The present findings contribute to this line of research by showing an information processing bias in relation to social anxiety to learn via avoidance strategies. As only social stimuli were used in the current study, it will be useful to assess whether these biases are restricted to contexts that are socially evaluative or whether they are generalizable to non-social contexts, as is the case in several psychiatric and neurological populations.

### Caveats to Bear in Mind

It is important to note that there are other probabilistic learning paradigms that are widely adopted in the literature, such as the probabilistic classification learning task (Gluck and Bower, 1988; Knowlton et al., 1994) and the Iowa Gambling task (Bechara et al., 1997; Brevers et al., 2013), which differ from the present probabilistic category learning task with regard to context, learning outcomes, probabilistic contingencies, task strategy, and so on. Studies using such paradigms have indicated that the probabilistic learning of emotional contents are hindered by heightened fear (Thomas and LaBar, 2008; Exner et al., 2014; Zetsche et al., 2015), such as in the case of obsessive compulsive disorder. However, as there has been little discussion thus far about the commonalities and distinctions between the properties of the different probabilistic paradigms, it is challenging to integrate such findings within the context of the current study. Other factors that differentiate between the tasks include the numbers of stimuli/cues used in the task as well as the parameters to determine the contingencies of the stimuli/cues. In the current task, the contingencies of each stimulus is incumbent on the other stimulus within the pair (A:B = 80:20, C:D = 70:30, E:F = 60:40). In contrast, in the probabilistic classification learning and Iowa gambling tasks, the stimulus/cue is independently associated with each outcome. Such factors together with other differences in stimulus type and task contingencies as well as type of sample under study render if difficult to compare the findings from our study with others that employ these alternative paradigms.

The present probabilistic category learning task allows us to examine whether participants are more sensitive to learn from reward versus punishment based on the relative dominance of the learned associations between the cues and the negative or positive outcomes. In contrast, in probabilistic classification learning and Iowa gambling tasks, participants are required to develop a strategy to best predict a negative outcome versus its absence, but it is not tested whether participants learn more from positive as opposed to negative reinforcement (but see Busemeyer and Stout, 2002; Ahn et al., 2014). Notably, highly fearful individuals (Thomas and LaBar, 2008) or those suffering from clinical disorder such as OCD (Exner et al., 2014) only demonstrated impaired learning when emotionally relevant cues were used within an emotionally relevant context. For example, participants were asked to predict what they would encounter when walking in the woods (Thomas and LaBar, 2008) or assume having to predict an epidemic as part of their job (Exner et al., 2014). Such contexts may induce situational anxiety that interferes with associative learning. Indeed, patients with social phobia were less likely to rely on optimal strategies in both a standard and an emotional probabilistic task using disease/contamination-relevant cues (i.e., no socially threatening materials), an effect which was mediated by situational anxiety (Zetsche et al., 2015). In the present study, except for using socially relevant cues and outcomes, no cover story was used to create a socially threatening context.

### Limitations and Future Directions

It would have been useful to analyze if the groups differed in terms of their learning curves in the acquisition of the different probabilistic contingencies in the training phase. This would require that the trials be discretely separated into several blocks such that the overall contingencies (i.e., A: 80% and B: 20 % positive reinforcement) are modeled within each of the blocks. As group differences were not expected within the training phase, a detailed block structure was not adapted in this study and only the overall contingency rate was set *a priori*. The learning curves could therefore not be assessed meaningfully and this constitutes one of the limitations of the study. One potentially promising avenue in this regard for future work would be to undertake computational modeling in order to estimate learning rates for reward and punishment (Frank et al., 2007).

It should be noted that other clinically relevant indices to assess the propensity for conditions that are often co-morbid with anxiety, such as depression and trait anxiety, were not recorded. It is therefore not possible to ascertain whether the pattern of findings might differ had other potentially co-morbid variables also been included in the analyses.

One potential interpretation of the findings of better performance on the part of the highly socially anxious group in the most ambiguous learning conditions is that this was likely to be moderated by a greater motivation to perform well as has been indicated in other studies (Alden et al., 2004; Roelofs et al., 2010). However, the present findings cannot be fully accounted for in this manner as the highly socially anxious group were no better in terms of overall performance in discriminating the different contingencies, and this would be expected if they were more highly motivated in general.

Although comparable to that of other studies in the field using a similar approach, the sample size of the study is modest and the sample is not ideally representative as it only includes undergraduates. These as such constitute methodological shortcomings of the study. In order to understand the clinical implications of these findings, further analogue studies of social anxiety will necessarily need to be accompanied by clinical investigations of instrumental learning in social anxiety disorder.

Another limitation of the study is that as only faces were employed as the learning and feedback stimuli, what cannot be ascertained from the analysis of the present findings is whether this tendency for a positive information processing bias or a greater sensitivity to actual reinforcement contingencies in instrumental learning is limited to particular contexts involving socially relevant stimuli and/or socially evaluative feedback, or whether it extends to non-social stimuli and/or non-social feedback. In fact, no investigation to date has employed both social and non-social variants of positive and negative feedback stimuli (verbal or non-verbal) within a single learning paradigm when assessing information processing biases as a function of social anxiety. Future studies that do so will be able to target the domain specificity of this propensity, which will be very beneficial in characterizing the dynamics of the cognitive mechanisms associated with social anxiety.

## Conclusion

An established probabilistic category learning paradigm was adapted using social stimuli in the present study to determine potential information processing biases in relation to social anxiety. Two key findings emerged. First, highly socially anxious people tended to be more accurate at determining actual probabilistic contingencies under ambiguous conditions. Second, the evidence suggests that the learning strategies used by the high social anxiety group were characterized by an enhanced avoidance of negatively reinforced stimuli. Assessing information processing mechanisms that underlie reinforcement learning in socially evaluative situations provides a promising avenue for future exploration in the context of social anxiety.

## Conflict of Interest Statement

The authors declare that the research was conducted in the absence of any commercial or financial relationships that could be construed as a potential conflict of interest.

## Footnotes

1 Please note that the fear of negative evaluation and social anxiety are not synonymous constructs. The fear of negative evaluation is the core symptom of social anxiety disorder or social phobia.

2 The rationale behind adopting this approach of classification includes (a) approximately normal distribution of screening sample scores, and (b) avoiding the problem of regression to mean (Preacher et al., 2005; Stevens et al., 2014).

3 Based on a sample of *n* = 43 as the data file with the individual FNE items of the whole sample was found to be corrupt, and we were therefore unable to access this particular data.

4 These exclusion criteria were developed to address the objectives of this study. There are no clearly established norms for rejecting participants in this task. Many studies do not mention which exclusion criteria, if any, were applied (e.g., Klein et al., 2007; Cavanagh et al., 2011a; Endrass et al., 2011) while others clearly impose criteria (e.g., Frank et al., 2004; Ott et al., 2011) but these parameters differ between studies and/or have been described with insufficient detail and/or are suboptimal with regard to establishing whether learning actually took place.

5 Please note that it is possible that some participants may have learned the task despite being 1.5 SD below the mean in accuracies. A stringent cut-off level was maintained to exercise caution in order to allow sound interpretations of the findings in this exploratory study.

6 As a check, all analyses were carried out using gender as the between-subjects factor. There were no significant gender-related differences (main and interaction effects) with regard to stimulus characterization, performance in the learning phase, and performance in the test phase (all *p* > 0.1).
